# Women physicians in cardiovascular magnetic resonance: Past, present, and future

**DOI:** 10.3389/fcvm.2022.984326

**Published:** 2023-01-04

**Authors:** Lilia M. Sierra-Galan, Niti R. Aggarwal, Jadranka Stojanovska, Subha V. Raman, Yuchi Han, Vanessa M. Ferreira, Katharine Thomas, Nicole Seiberlich, Purvi Parwani, Chiara Bucciarelli-Ducci, Lauren A. Baldassarre, Sophie Mavrogeni, Karen Ordovas, Jeanette Schulz-Menger, W. Patricia Bandettini

**Affiliations:** ^1^Cardiology Department of the Cardiovascular Division at the American British Cowdray Medical Center, Mexico City, Mexico; ^2^Department of Cardiovascular Disease, Mayo Clinic, Rochester, MN, United States; ^3^New York University Langone Health, New York, NY, United States; ^4^Indiana University School of Medicine, Indianapolis, IN, United States; ^5^The Ohio State University Wexner Medical Center, Columbus, OH, United States; ^6^University of Oxford Centre for Clinical Magnetic Resonance Research, Oxford British Heart Foundation Centre of Research Excellence, The National Institute for Health Research Oxford Biomedical Research Centre at the Oxford University Hospitals NHS Foundation Trust, Division of Cardiovascular Medicine, Radcliffe Department of Medicine, University of Oxford, Oxford, United Kingdom; ^7^Department of Radiology, University of Michigan, Ann Arbor, MI, United States; ^8^Division of Cardiology, Department of Medicine, Loma Linda University Health, Loma Linda, CA, United States; ^9^Royal Brompton and Harefield NHS Foundation Trust, London, United Kingdom; ^10^Section of Cardiovascular Medicine, Yale School of Medicine, New Haven, CT, United States; ^11^Onassis Cardiac Surgery Center, Athens, Greece; ^12^Department of Radiology, University of Washington, Seattle, WA, United States; ^13^Charité - Universitätsmedizin Berlin, Corporate Member of Freie Universität Berlin, Humboldt-Universität zu Berlin, ECRC Cardiology, Helios-Clinics Berlin-Buch, Clinic of Cardiology and Nephrology, DZHK Partnersite Berlin, Berlin, Germany; ^14^National Heart, Lung, and Blood Institute, National Institutes of Health, Bethesda, MD, United States

**Keywords:** women, cardiovascular MRI, cardiovascular imaging, gender diversity, leadership, CMR, inclusion, equity

## Abstract

Women’s engagement in medicine, and more specifically cardiovascular imaging and cardiovascular MRI (CMR), has undergone a slow evolution over the past several decades. As a result, an increasing number of women have joined the cardiovascular imaging community to contribute their expertise. This collaborative work summarizes the barriers that women in cardiovascular imaging have overcome over the past several years, the positive interventions that have been implemented to better support women in the field of CMR, and the challenges that still remain, with a special emphasis on women physicians.

## Introduction

Women have long struggled for equal footing in many aspects of society. While, in recent years, their unique value in some areas traditionally dominated by men has become better understood, women still work to achieve recognition in many professional fields. Expressly, women in medicine often have not been granted the privileges that are afforded to men with the same degrees and training. In cardiovascular imaging and cardiovascular MRI (CMR), in particular, the challenges faced by women make it difficult for them to enter the field and reduce their likelihood of staying.

The first barrier to entry in the field of cardiovascular imaging is the long and challenging training, consisting of medical school and residency (in either diagnostic radiology or internal medicine), followed by cardiology and dedicated cardiac imaging or cardiovascular fellowships. Those who choose to engage in cardiovascular imaging research, either as a scientist pursuing a Ph.D. or a physician-scientist, must take on additional training. This lengthy process may cause women to hesitate when making the decision to pursue a career in cardiovascular imaging. The long career process is a gender-neutral consideration; however, in parallel, in the current era, women in some societies bear more responsibility for the family. As such, they must be capable of contributing more substantially to the support of their families and the inevitable family circumstances, including birth, death, divorce, relocation, and consequences of financial troubles. The long educational process before becoming fully-trained in cardiovascular imaging, along with the gender pay gap, may contribute to these obstacles.

Once this training is completed, women may still face challenges in clinical cardiovascular imaging practice. In many countries, cardiology and radiology have traditionally been viewed as “male” specialties, making it difficult for women to excel (if they even select these specialties in the first place). Among CMR experts, more men hold tenured academic positions than women (68% vs. 32%).^1^ As a result, women are outnumbered by men in the fields of cardiology and radiology (1). Although institutions may seemingly accept the female presence in their respective departments, they may still underappreciate the contributions of women colleagues and are more likely to appoint men to leadership positions.

These lower numbers of women physicians in fields such as cardiovascular imaging may reduce the quality of care offered to patients, as the presence of women in multi-disciplinary teams has resulted in improved outcomes measured by lower patients’ 30-day mortality and readmission rates (2, 3). Modern medical practice requires many individuals with different areas of expertise, and the advanced imager is an essential part of the team taking care of cardiovascular patients. Advanced imaging using approaches including cardiovascular magnetic resonance (CMR) has revolutionized the field of cardiology with high spatial resolution and three-dimensional capabilities. CMR is now indicated for various cardiovascular conditions, with extensive evidence available to showcase its impact on overall outcomes and clinical practice (4, 5). Unfortunately, as described above, women in the field of CMR—among other subspecialties—face the challenge of balancing personal life, family responsibilities, and career demands. Specialized training inherent to CMR adds to this challenge. Unfortunately, only a few dedicated centers provide advanced CMR training, the competition is hard, and a strong biographic sketch is necessary to be accepted.

This manuscript is meant to provide an understanding of the current state of women in cardiovascular imaging and CMR and to suggest some practical changes that could be enacted to help recruit and retain women in CMR. To this end, the historical role of women in cardiovascular imaging, the challenges women face in CMR, the current state of the field by the numbers, the importance of the presence of women in CMR, and the potential solutions to these challenges are discussed. While the importance of women in all aspects of CMR is acknowledged, this work is meant primarily to highlight those challenges faced by female cardiologists and radiologists in the field of cardiovascular imaging.

## History of women in CMR

Women have played an essential role in the development of medical imaging. A prime example is Marie Curie, an early pioneer of “in-the-field” medical imaging. While best known for her research on radioactivity, Professor Curie was pivotal in implementing mobile x-ray units during World War I (6).

Compared to x-ray, cardiovascular magnetic resonance (CMR) is a relatively recent addition to the medical imaging toolbox, with its origins in the early 1980s. However, women were among the early developers of this emerging technology. For example, Dr. Joanne Ingwall was instrumental in promoting cardiovascular spectroscopy (7) and served as the first female president of the International Society for Magnetic Resonance in Medicine (ISMRM). In the 1990s, Daisy Chien, Ph.D., developed MRI pulse sequences for cardiovascular imaging, including magnetic resonance angiography, spin-echo T2 MRI for the detection of acute myocardial infarction, perfusion imaging, and left ventricular segmental TrueFISP imaging (8–11). Her scientific status led to a position on the ISMRM Board of Trustees. Katherine Wu, M.D. demonstrated microvascular obstruction as a predictor of adverse outcomes. Her work using CMR to predict sudden cardiac death outcomes helped push CMR into mainstream clinical use (9). Similarly, Brigitte Poncelet-Belliveau, Ph.D., developed a broad spectrum of CMR sequences and applications, including blood oxygen level-dependent contrast (BOLD), echo planar imaging, myocardial perfusion, and T2-TrueFISP (12–14). In the 2000s, Dr. Vivian Lee, a radiologist and ISMRM Gold Medal Winner, developed the MRI research program at New York University. She worked to improve methods of assessing vascular disease with 3D gadolinium MRA and non-contrast methods. She also developed CMR viability imaging (15–17) and served on the Board of Trustees and later as President of the ISMRM.

By the early-to-mid 2000s, an increasing number of women from many fields (physicists, cardiologists, radiologists, and technologists) had entered the field of CMR. Critical work in translating the CMR methods into cardiovascular applications was carried out by key individuals, such as Jeanette Schulz-Menger, M.D. with her work in inflammatory disease and myocarditis (18–20)—and the first female president of the Society for Cardiovascular Magnetic Resonance (SCMR), as well as a member of the ISMRM Board of Trustees, Subha Raman, M.D., the second female president of the SCMR, with her innovative treadmill CMR research (21), Chiara Bucciarelli-Ducci, M.D.’s—the second chief executive officer (CEO) and first female CEO of the SCMR—contributions to better understanding myocardial infarction in the setting of non-obstructive coronary arteries (MINOCA) (22) and many other women imaging experts. While CMR has become more widespread over the past 15 years, new methods are continuously being developed, validated, and deployed for patient care through integrated technological, translational, and clinical development.

## Barriers to entering and remaining in the CMR field

Professional women in all careers face layered challenges. CMR is a physically and mentally demanding and fast-paced specialty. Here we describe some aspects of CMR that deter women from entering the field and the challenges of remaining engaged in the field. Although the field of CMR benefits from multi-disciplinary collaboration between specialists in cardiology, radiology, and physics/engineering, these different groups face different obstacles. While many similar challenges are experienced by women scientists in cardiovascular imaging, portions of this section are more applicable to women physicians, as the pathways for cardiologists and radiologists overlap more than that of the technical experts.

### Absence of female physicians

Historically, women were not allowed to train as doctors. As a result, parity in medical school intake has only been achieved in the last few decades (23, 24) with some countries continuing to lag (25). Fortunately, this recruitment barrier appears now largely resolved, with medical school graduates comprising an equal number of males and females. Improved female representation of women in medical schools is promising for increased representation of women in cardiology, diagnostic radiology, and cardiac imaging.

Even with this growing number of women in medical schools, it is interesting how medical professionals may mistakenly infer that women are now broadly well-represented, overestimating the true representation. The misperception could produce growing reservations or less support for gender equality initiatives and political support (26).

In reality, there is a progressive decline in female representation from each training step to clinical practice in cardiology. In the United States, women comprise 51% of medical school graduates and 43% of internal medicine residents, yet they are underrepresented in cardiology training and practice (27). Currently, 12–28% of cardiology trainees (23, 24, 28) and 24–30% of radiology trainees are female (29). In comparison, only 12–14% of fully trained cardiologists are women. Worldwide, women are less likely to reach the highest levels of cardiology (23, 24, 27, 28, 30–32). Though some of this disparity will inevitably improve as current trainees complete their training, the attrition rate remains high.

Literature reports that many women have been actively discouraged from becoming cardiologists. For example, numerous female cardiologists were told as young doctors not to become cardiologists because they are female (33) and sometimes because they were too “nice,” an assertion male doctors rarely encounter. Women also report being denied consultant, i.e., attending, cardiologist jobs, as other consultants would not work with women (33). In addition, although challenging to quantify formally, many women experience discrimination when applying for medical training posts or other career opportunities during their reproductive years, as it is often perceived that they may become pregnant and unavailable for clinical duties and call schedules. A male candidate is thus often preferred. When faced with these attitudes, many women will simply choose a specialty that demonstrates that they want them.

### Work–life balance and the risk of burn-out

The demands of cardiology make an appropriate work-life balance difficult to maintain. Women looking to choose their specialty often consider this aspect carefully and are more likely than men to value family-friendly specialties, female-friendly specialties, and stable hours (34). Prospective trainees see the reality of life as a cardiologist: 38% of prospective female cardiology trainees report their female mentors not having a reasonable work-life balance (35). There is also the issue of long hours: in Japan, 50% of female cardiologists work more than 960 h of overtime a year, with more than 60% considering leaving the field due to gender discrimination, pregnancy, and children (36). Once within the cardiology specialty, trying to maintain a reasonable work-life balance can hinder training and career opportunities (35).

Similarly, for radiology, the rigors of rotations, an ever-increasing workload, and call schedule make at least half of the female radiology trainees and junior faculty prone to burn-out stemming from a poor work-life balance (37). In fact, burn-out is more prevalent among female radiology trainees entering parenthood because the radiology and cardiothoracic radiology training and early junior faculty years occur during the prime childbearing ages (38). Given this inference and the demographic shift of increasing mean childbearing age, radiology trainee-parents become a minority, with only 21% having one or more children (39). Additionally, 27% of radiology trainees are women likely experiencing work-life imbalance during early motherhood, thus precipitating burn-out among female diagnostic radiologists, making the specialty unattractive to women.

In an American College of Cardiology (ACC) life survey, female cardiologists and cardiology trainees had a 7% higher burn-out prevalence than male peers (40). Based on a Medscape survey of physicians from June to September 2021, radiology was the seventh most common medical specialty to be associated with burn-out (49%), with the highest level occurring among women compared to men (65% vs. 44%) (41). This results in low self-esteem, decreased career satisfaction, social dysfunction, poor well-being, and inevitable attrition from the profession.

### Lack of female role models

Despite improvement over the decades, there remains a paucity of female role models and mentors within cardiology. Women cite a lack of female role models that creates hesitancy to apply for cardiology specialty training (34). However, simply being able to see that female cardiologists exist and succeed may be sufficient to encourage young women to pursue cardiology training (42). The value of female role models for diagnostic radiology and engineering sciences also holds true.

### Discrimination, harassment, and bullying

Women face discrimination, bullying, and harassment more frequently than men. Discrimination occurs across the world and is reported by female cardiologists 62% of the time in the UK, 65% in the US, and 68% worldwide. In the US, this figure has changed little over the past two decades (31, 43–45). Examples of gender-based discrimination include women not being introduced by their professional titles, patients transferring their care to male colleagues, and an implicit assumption by some men that women are simply not up to the pressures of cardiology with a corresponding loss in career opportunities (46, 47).

Regarding bullying, surveys of both trainees and consultants in the UK found that women were significantly more likely to have experienced bullying than men, and this bullying was usually sexist in nature. Women also report high levels of sexual harassment (36% of British and 12% of worldwide female cardiologists) (43, 44, 48).

Women may turn to senior female role models to seek advice to deal with these problems; however, the limited number of women in leadership of institutions, professional societies, and editorial boards limits availability. Additionally, many of these female role models face impossible pressures to succeed themselves and, at times, may adopt a more stereotypically masculine behavior and shun other women, keeping solidarity with their male peers (49). Improved networking among women professionals might provide another level of support.

### Family planning

Female cardiologists frequently express concern about how to plan their family while also having a career (34). This is not helped by perceived unfriendliness from their employer—43% of female cardiologists in the US have been asked about family planning in an interview setting.

Female cardiologists are less likely to be married (75% women vs. 89% men) and less likely to have children (72% women vs. 87% men). Many factors contribute to this discrepancy: in addition to institutional hostility, women who adopt part-time work patterns or take more extended periods of maternity leave are frowned upon and miss out on opportunities at work (50). Female cardiologists are also more likely to require paid childcare, whereas male cardiologists often have spouses that care for their children (46, 51). The outdated yet prevalent cultural norm of the female shouldering most of the childcare burden appears to prevail even when the female is the primary salary earner.

Maternity leave policies differ significantly worldwide. In the US, an outlier in parental leave policies, 50% of female cardiologists took eight weeks or less of maternity leave, with only 3% taking more than 6 months. One-quarter reported that their maternity leave was unpaid, and more than half felt pressured to return to work early. Cardiologists in training felt particularly pressured compared to those that had completed training. One-third of female cardiologists in the US also reported being asked to do extra service or call prior to their maternity leave (50, 52).

Maternity leave of female cardiologists outside of the US has not been studied in as much detail as in the US; however, in a worldwide survey of female surgeons (a similar cohort to female cardiologists), average maternity leave was between 7 and 12 months. However, in this worldwide survey, only half felt that their employer was generally supportive, and 80% of female surgeons reported being told that a surgical career was incompatible with parenthood (53).

### Radiation exposure

Likely for safety reasons, pregnant people were often excluded from any place where radiation exposure could occur. While CMR *per se* does not involve radiation, advanced imagers often train in both CMR and cardiovascular computed tomography (CCT), using X-rays for imaging. More recently, this level of extreme caution around pregnant people has been replaced by warnings coupled with more monitoring. For example, there are now guidelines regarding radiation exposure that set strict limits and include using a fetal monitoring badge (54, 55). These guidelines are not overly restrictive and allow training and career progression to continue even when pregnant safely. Despite this added vigilance, women are increasingly more concerned about radiation exposure than previous cohorts (50, 51, 54, 55). This concern may potentially result in less exposure to training or scanning involving radiation and fewer opportunities due to this reduced experience.

### Responsibilities outside of work

With arduous long hours, overnight calls, and at times, a competitive, cutthroat work environment, the fields of radiology or cardiology are often not conducive to family life, discouraging women from considering these fields as a career. As mentioned, female cardiologists often have more domestic responsibilities than their male counterparts, spending around 8.5 h more on household tasks per week than their male peers. They also shoulder more caretaking—for sick children and parents in need—compared to their male counterparts (56, 57). With average work weeks of 40–60 h, these extra responsibilities outside of work can quickly create unsustainable pressure (58). Flexible work patterns can help, but these can be difficult to negotiate and achieve (23, 51). Part-time working patterns are often unavailable to trainees, who are most likely to have very young children. Where part-time work is allowed, there is still very low uptake: only 4% of cardiology trainees in the UK work part-time (59). Once fully trained, working part-time is also uncommon: only 10% of female cardiologists in the UK work part-time as opposed to 4% of men (44). Part-time work brings its own challenges: lower pay, fewer career opportunities, and loss of status. A recent British survey showed that cardiologists who work part-time are perceived as having lower status (44).

### Academia

Women are underrepresented in cardiovascular academia: only 17% of faculty appointments are women. Women have significantly lower rates of the first authorship, particularly in high-impact journals, and are cited less often (60–63). Female cardiologists are often neither involved in the high-impact trials nor on the writing committees of clinical guidelines (64–66). Women in academia also have less success in career development awards, with gender differences persisting in a number of awards for clinician-researchers in the US, even when adjusting for confounders (67). Similarly, women in radiology are under-represented, with only 34% of women joining academia in the US. However, this number declines at higher levels of leadership, reaching just 25% among section chiefs and vice chairs and 9% among chairs (68).

### Salary

Significant gender inequity persists when it comes to compensation. Female cardiologists continue to earn, on average, $32,000 less than male cardiologists in both private practice and academia, even after controlling for location, subspecialty, and full-time status (65, 66, 69). This disparity in earnings is exacerbated by the cost of children, both in fewer work hours or hired child care: either women are predominantly responsible for looking after their children (and thus not able to work and earn), or they must arrange paid childcare, which will cost around 11–20% of their salary (44, 53, 56).

### Political dimension

As mentioned above in their independent sections, societal habits and traditions, such as family planning, marriage, divorce, relocation, work-life balance and the risk of burn-out, lack of female role models, discrimination, harassment, and bullying, radiation exposure, responsibilities outside of work, academia and salary, among others, where women are submitted to more substantial constraints than men, have reached a political dimension in some countries in the world, leading to action and legislation specifically to help women with these aspects for better professional equity (70).

## Current CMR practice in the world

**FIGURE 1:**
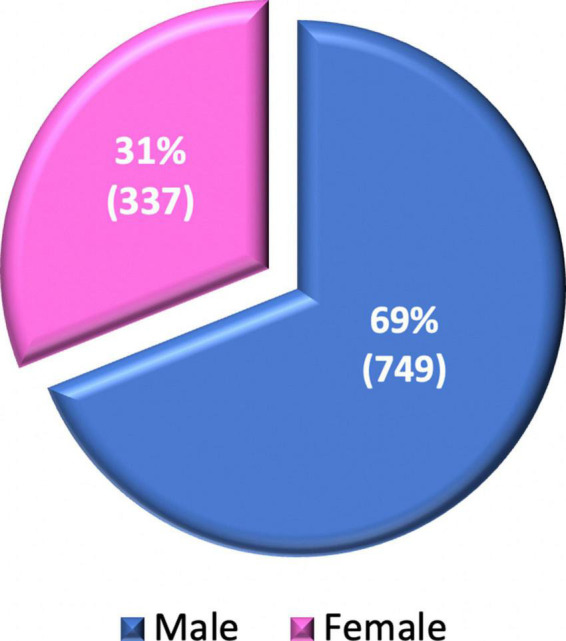
Gender distribution of CMR surveyed practitioners.

Utilizing the data obtained by a survey launched by the Society for Cardiovascular Magnetic Resonance (SCMR) in 2017 that is currently under review by the Journal of Cardiovascular Magnetic Resonance (see text footnote 1), an analysis was performed to identify women’s participation in CMR practice around the world. Of 1,086 respondents, 337 (31%) were women, as shown in Figure 1.

**FIGURE 2:**
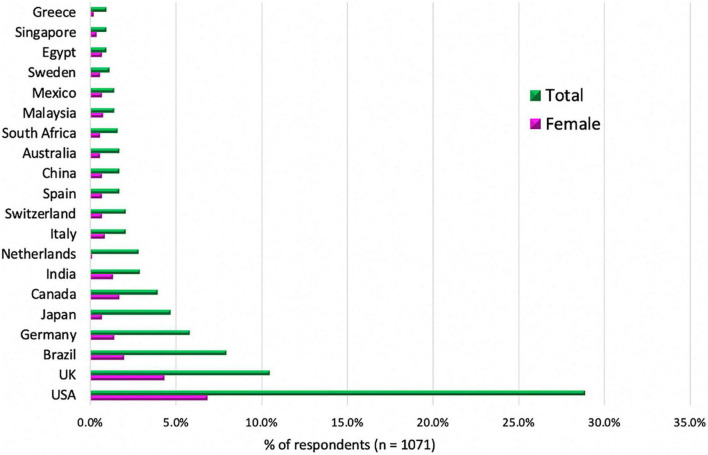
Top 20 countries of origin of surveyed respondents (*n* = 1,071). The top 20 countries with the most respondents are shown. The data from all other respondent countries are not displayed in the graph. The detailed description of the responders and their gender (within parentheses) were as follows, for USA 309 (F:73/M:236), UK 112 (F:46/M:66), Brazil 85 (F:21/M:64), Germany 62 (F:15/M:47), Japan 50 (F:7/M:43), Canada 42 (F:18/M:24), India 31 (F:14/M:17), the Netherlands 30 (F:1/M:29), Switzerland 22 (F:7/M:15), Italy 22 (F:9/M:13), China 18 (F:7/M:11), Australia 18 (F:6/M:12), Spain 18 (F:7/M:11), South Africa 17 (F:6/M:11), Malaysia 15 (F:8/M:7), Mexico 15 (F:7/M:8), Sweden 12 (F:6/M:6), Egypt 10 (F:7/M:3), Greece 10 (F:2/M:8), Singapore 10 (F:4/M:6). The rest of the surveyed countries are described in the Supplementary material.

The percentage of female CMR practitioners who responded to the survey varied significantly depending on the geographical location (Figure 2). Female respondents were as follows: in New Zealand, Thailand, Romania, Indonesia, Egypt, Uruguay, and Kuwait, women represented 67–80% of respondents; in Norway, Malaysia, Sweden, Hong Kong, Lithuania, Algeria, Republic of Korea, and Finland, 50–57% of respondents were women; in Mexico, India, Canada, the UK, Italy, Singapore, Denmark, Spain, China, South Africa, Australia, Argentina, Colombia, Austria, South Korea, Chile, and Switzerland, 32–47% of respondents were women; in Saudi Arabia, the Czech Republic, Brazil, Germany, and the US, Greece, and Hungary, only 20–25% of respondents were women; in Portugal, Ireland, and Japan, 14–17% of respondents were women; and only 3% of the Netherlands respondents were women.

In the Philippines, El Salvador, Nicaragua, Iran, Georgia, Russia, Panama, Myanmar, Morocco, and Monaco, there were 1–3 respondents, and all of them were only women.

There were no responses from Turkey, France, Belgium, United Arab Emirates, Qatar, Bangladesh, Slovakia, Poland, Pakistan, Ecuador, Andorra, Venezuela, Oman, Mongolia, Lebanon, Vietnam, and Kazakhstan women.

**FIGURE 3:**
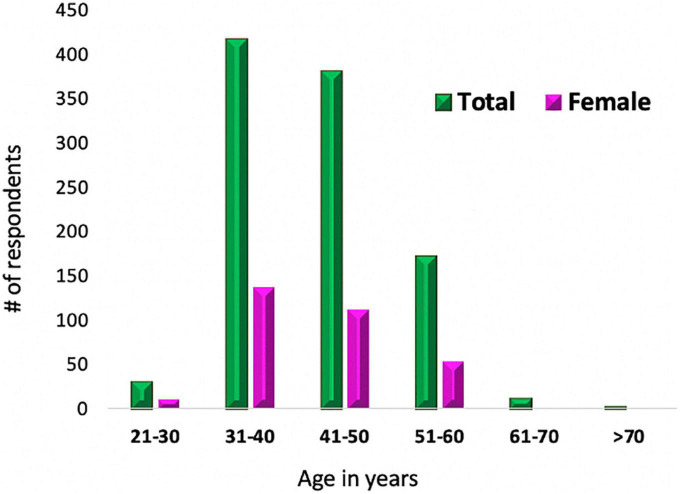
Age distribution of surveyed CMR practitioners. This graph displays the age range distribution of the CMR surveyed practitioners with the breakdown of the percentage of women respondents.

The age distribution by ranges showed almost a consistent trend of 30% of women respondents for ages less than 60 years; women made up a smaller percentage of older respondents (8% in the range of 61 to 70 years old, and 0% older than 70 years). This is concordant with the history of CV imaging and CMR, mainly dominated by males in the past. However, an exciting and encouraging fact revealed is that the highest percentage of women respondents was in the youngest surveyed range (from 21 to 30 years old), demonstrating an apparent increase in women joining the field in recent years (Figure 3).

**FIGURE 4:**
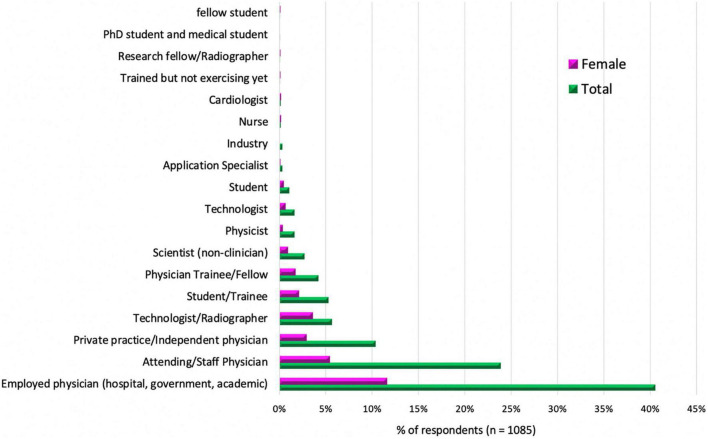
Stage of training or primary practice type of the CMR survey practitioners. This bar graph shows the different types of primary practice or stages of training of those CMR surveyed practitioners. The distribution is shown in percentages of total (male and female) in green and just female respondents in pink.

**FIGURE 5:**
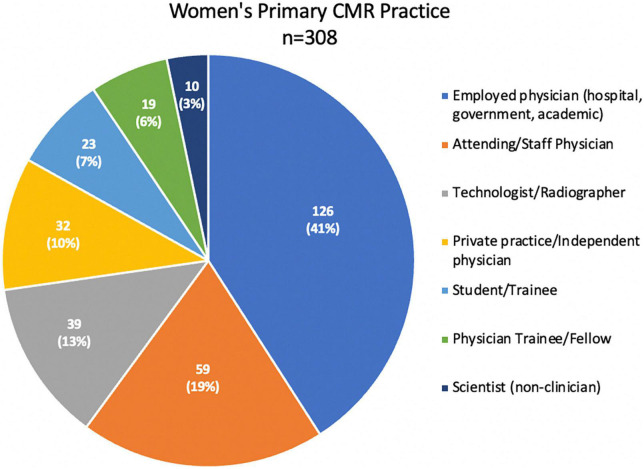
Women’s primary CMR practice of the CMR survey practitioners. Responses in this category = 308. This pie graph shows the distribution of the women’s primary CRM practice as employed physicians at different kinds of institutions such as academic, community, or government hospitals (*n* = 126, 41%), attending or staff physicians (*n* = 59, 19%), technologists or radiographers (*n* = 39, 13%), private practice or independent physicians (32, 10%), pre-grade students or trainees (23, 7%), physician trainees, residents or fellows (19, 6%), and scientist (non-clinician) (10, 3%).

When examining the most common practice types of those working in CMR, it is interesting to note that for both men and women, there is more CMR practice in the academic and government scenarios compared to private practice, as displayed in Figures 4, 5. One might speculate that the economics of performing, interpreting, and reimbursement of CMR may contribute to this difference. However, there may also be less representation of private practices and small private hospitals within the SCMR; thus, the data should not be overinterpreted.

## Recruiting and retaining women in cardiovascular imaging

Both recruiting women and retaining women physicians and scientists in cardiovascular imaging are essential to increasing their numbers. Progress is slow, but it is also encouraging: the proportion of women is increasing in medical school and in cardiology and radiology training programs (23, 27). Likewise, the number of women physicists and engineers is growing. However, over the past decade, the percentage of women in diagnostic radiology training has remained steady at 30% (29, 71). As women’s numbers and leverage increase, they will begin to assert themselves to negotiate more favorable conditions: good parental leave policies, flexible work patterns, and equal pay (51, 72). These should be available for both women and men to help eliminate tendencies to avoid hiring female candidates. Those running training programs should consider optimizing work conditions for everyone to continue attracting the best candidates.

To increase the number of women entering cardiology, diagnostic radiology, and cardiac imaging, it is essential to target women interested in medicine or a biomedical career early in the process while in high school, college, or medical school. Additionally, there are too few mentors targeted explicitly toward working with women. Imaging societies must provide avenues for trainees and early career professionals to interact with expert advanced cardiovascular imagers. In addition to increasing the number of mentors from both genders, educating and engaging those in leadership about gender disparities and biases are essential. Professional societies should create initiatives to ensure that diversity is a priority and that women advanced imagers are supported to increase representation. This should happen both at the level of societal leadership, societal committees, and core groups.

Despite rising awareness of women’s unique challenges, the need for flexible working patterns, and better family planning policies, barriers remain. The recent global pandemic has shown how quickly progress can be eroded: women have faced a disproportionate impact, taking on increased domestic and childcare responsibility, affecting jobs and salaries (73, 74).

The early career remains a challenging period for female professionals due to the tension between job requirements and additional family responsibilities, including childbearing. Supporting female physicians and scientists during this difficult time by instituting policies for maternity leave and ensuring flexible work hours (including part-time positions, telework, and different adaptable strategies) can help retain women as advanced imagers in the field. The increasing burn-out rate among female physicians should serve as an impetus for many institutions to adopt and support physician-parent wellness, such as a prolonged family leave policy and sustaining gender diversity and parity in advancements and leadership for female faculty. Establishing and supporting groups, such as women in radiology or parenting mentorship, will pay dividends in the form of improved retention of female faculty and well-being. This premise is based on the literature that radiology trainees and junior faculty reported increased networking (94%) compared to senior faculty (69%) and increased research involvement, which accelerated the professional development and contributed to a more diverse and enabled workforce (75).

Institutions and professional societies must enforce a no-tolerance policy for sexual harassment or bullying in the workplace. Institutional leadership’s responsibility is to create a culture to promote a safe environment where victims of sexual harassment and gender bias feel empowered to report it.

Additionally, institutions should regularly conduct reviews and analyses of faculty salaries to uncover any unconscious biases in salary negotiation.

**FIGURE 6:**
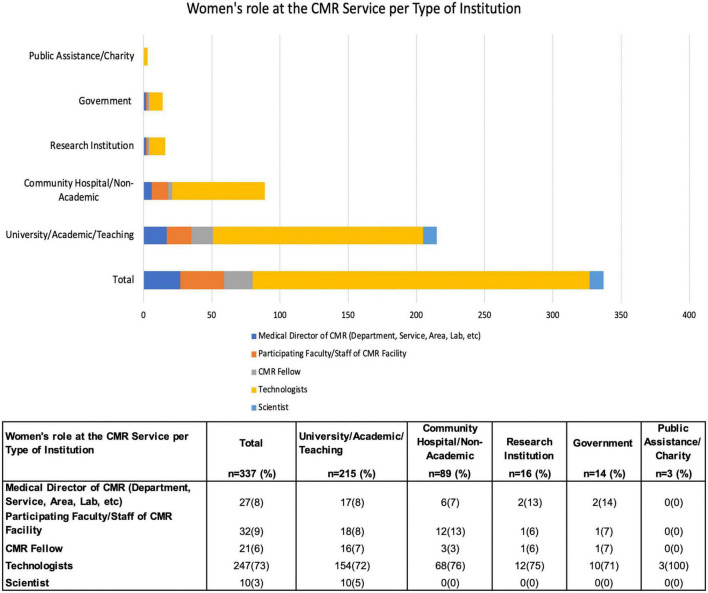
Women’s role at the CMR Service per Type of Institution of the CMR survey practitioners. Responses in this category = 337. This stacked bar graph shows the women’s role at the CMR service per type of institution, where 64% practice in the university, academic, or teaching environment. Although it is where women primarily participate in leadership roles, 26% of women develop their professional activities in the community or non-academic institutions, where 76% participate as technologists. Women scientists are working only in universities or academic institutions. Detailed data shown in the graph description is in the table at the bottom of the figure.

The empowerment strategies in cardiovascular imaging parallel women’s empowerment in cardiology, diagnostic radiology, and physics/engineering, which include increasing opportunities for leadership training and women in leadership positions who can serve as role models such as advocates, coaches, and mentors for other women (Figure 6).

To improve retention rates of women in cardiology, societies such as the British Cardiac Society (BCS) and American College of Cardiology (ACC) have created Women in Cardiology (WIC) groups that serve as important leadership, career development, and advocacy forums for female cardiologists (76). In addition, the ACC has introduced several initiatives, including courses such as “Upping your game – clinical trials training,” aimed at providing opportunities for learning and networking for underrepresented minorities and women in the field. Furthermore, the Association of American Medical Colleges (AAMC) offers a “Mid Mid-Career Women Faculty Leadership Development Seminar” every year for mid-career women faculty who have been at the associate professor level for at least two years with “the knowledge and skills necessary to support their continued progress along the path to leadership in academic medicine and science.” The seminar includes organizational leadership topics and career-advancing strategies considered highly important for effective leadership throughout “various mission-critical activities.”

Some institutions have programs to mentor and promote women faculty. For example, at the University of Pennsylvania, there is a program called FOCUS (77) that focuses on the health and leadership of women with separate sections for medical students, residents and fellows, junior faculty, and senior faculty. This program addresses the key national issue of the underrepresentation of women in senior levels of academic medicine. Moreover, with the support from the Dean of the Medical School, FOCUS launched initiatives including seminars, workshops, and conferences related to career development and mentoring; faculty research seed grants and recognition awards, and medical student fellowships in mentored projects involving women’s health research to recruit, retain, and promote female leadership. These kinds of institutional and medical societal programs are crucial to providing leadership training and empowerment for female academic physicians.

Similarly, on the radiology side, there is a very active American Association for Women Radiologists (AAWR) that educates and enhances the professional fulfillment of female radiologists. In addition, almost every academic radiology department in the United States has adopted and supported women in radiology groups providing bona fide opportunities for mentorship and leadership growth (75). The Radiological Society of North America (RSNA) has embraced several committees empowering women across subspecialties. One such example is the Committee for Diversity, Equity, and Inclusion (DEI), whose member efforts are geared toward increasing the visibility of women in the field. For example, 57% of RSNA committee chairs are women; nine women have served on the RSNA Board of Directors, and seven women have served as RSNA President. Recognizing the need for women’s empowerment in radiology, the AAWR was formed 25 years ago to promote, educate, and advocate for women radiologists. The AAWR holds regular meetings and workshops tailored to meet women’s needs in radiology.

While there are no explicitly tailored programs/workshops for women empowerment in the cardiovascular imaging subspecialty, the North American Society of Cardiovascular Imaging (NASCI) has worked diligently to increase the representation and visibility of women within the society. In terms of women’s reputation in leadership (as tabulated below), NASCI follows closely with the Society for Cardiovascular Magnetic Resonance (SCMR). Table 1 (78–89) shows a current year comparison of the women’s participation in main US-based international cardiovascular imaging societies, using the leadership roles in executive officer positions and committees chairs as a surrogate of female involvement in the field. Note that the comparison is limited by what was available on each society’s publicly available website and differences between each organizational governance rule and represents more of an “at-a-glance” assessment of women’s representation in these societies. The American Society of Echocardiography (ASE) seems to be particularly successful in elevating women to leadership roles.

**TABLE 1:** Female leadership participation in the major US-based cardiovascular imaging societies.

	ASE [Ref (76–78)]	ASNC [Ref. (82, 83)]	NASCI [Ref. (79–81)]	SCCT [Ref. (84, 85)]	SCMR [Ref. (86, 87)]
**Presidents over the past 10 years** No. of women (% women)	4 (40)	1 (10)	3 (30)	1 (10)	2 (20)
Current executive officers** No. of women/#positions (% women)	4/8 (50)	2/7 (28.5)	3/6 (50)	1/7 (14)	2/5 (40)
**Current committee chairs** No. of women/#positions (% women)	9/18 (50)*	4/20 (20)	8/19 (42)	4/10 (40)	8/18 (44)

ASE, American Society of Echocardiography; ASNC, American Society of Nuclear Cardiology; NASCI, North American Society for Cardiovascular Imaging; SCCT, Society of Cardiovascular Computed Tomography; SCMR, Society for Cardiovascular Magnetic Resonance.

*These data reflect what was reported on the specific society’s public website on 26 June 2022.

**The “Executive Officers” varied slightly between societies but most often included a president, vice-president, treasurer, secretary, and past-president, among other roles.

Although women are well-represented in NASCI, gender parity has not been reached in all reaches of the society. The number of women speakers and moderators (*n* = 35; 33%) at the 2021 annual meeting indicates that we need to understand women’s needs better and increase their engagement in future meetings. These findings are reproduced at other organizations’ meetings. Improved support for women’s engagement may include providing childcare during the meeting, lactation rooms, work and life integration workshops, one-on-one mentor-mentee sessions, and/or short-term coaching sessions, which describe how to navigate through the system to offset any future decline of leadership role or active participation during the annual meetings.

From the highest leadership perspective, women are still underrepresented in imaging, as shown in Table 1. In addition, the percentage of female presidents of the imaging societies was lower than its female membership. However, in the executive officers (defined differently among societies but typically in addition to the president, including positions like the vice president, treasurer, and secretary, among other offices) and committee chairs, there is a higher representation of women than traditionally seen. To increase female leadership at the top, a pipeline of leaders must be developed through leadership education and mentoring. Both the American Society of Echocardiography (ASE) and the American Society of Nuclear Cardiology (ASNC) have leadership programs, although not explicitly geared toward women.

Recently, in 2020, The European Association of Cardiovascular Imaging (EACVI) formed its Task Force of Women in CV Imaging. The task force represents an initiative within the EACVI to connect members interested in promoting women’s representation both in the career setting and leadership roles and in clinical research development in CV diseases in women. This task force intends to unite women in CV imaging worldwide and provide opportunities that may not be available to all women professionals. The main goal intends to help low-to-middle-income country women imagers train within a European country with strong CV imaging expertise with a plan to then return to their home country and serve the community, providing continuous support by addressing challenging cases via remote communication (88).

**FIGURE 7:**
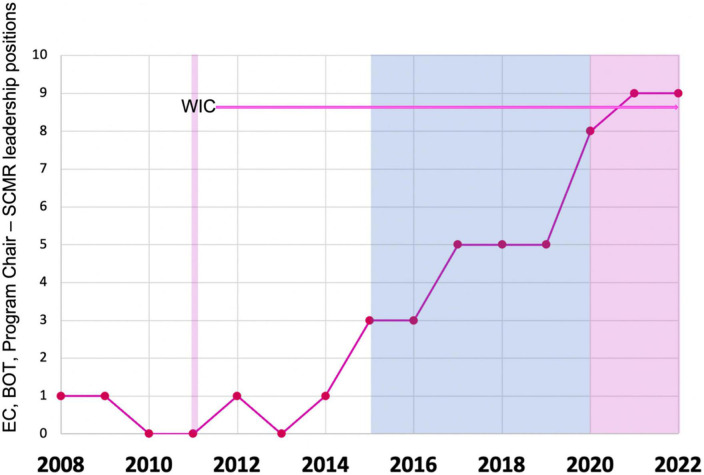
Women’s representation in SCMR leadership. The blue background represents the first CEO (male) and the pink the second and current CEO (female) of SCMR. The thick pink line marks the Women in CMR (Special group of SCMR) establishment as a Task Force by Dara L. Kraitchman, Ph.D., and supported by the E.C. leadership by Victor A. Ferrari, MD. EC, Executive Committee; BOT, Board of Trustees; WIC, women in CMR (Special group of SCMR).

The increasing involvement of women in the SCMR also corresponds to the initiation and sustained activities of the SCMR Women in CMR Group, which was the brainchild of Dr. Dara Kraitchman (Figure 7). The Women in CMR group meets at the annual SCMR Scientific Sessions and throughout the year. Since the advent of this group, a steady increase in women’s engagement in leadership positions within the SCMR over the past decade has been evident (Figure 7). In addition, women leaders have also stimulated and founded new interest groups like those focused on cardio-rheumatology and cardio-oncology, as well as the CMR translation working group that has advocated for the translation of key CMR documents into other languages to promote international dissemination of the field. These data indicate a shift in thinking and demonstrate positive support for women’s engagement within the CMR community. Intersocietal joint activities (e.g., between the SCMR and ISMRM or other imaging societies) may help to amplify awareness of women’s issues within the field of CMR and imaging, as well as help to jointly work on action items to solve some of the ongoing challenges.

## How CMR has made an impact on women’s cardiovascular health

Even as women have become more involved in the field of CMR, CMR has likewise impacted women’s cardiovascular health (90). Among the cardiovascular diagnoses affected are myocardial infarction in the setting of non-obstructive coronary arteries (MINOCA), small vessel disease ischemia, ischemic heart disease in general, rheumatological disorders, and cardio-oncology.

Historically, women with acute coronary syndrome exhibit symptoms that differ from men. Chest pain may be vague or completely absent. Women may present with fatigue or shortness of breath as a primary symptom. In the past, when women presented with an acute coronary syndrome with elevated cardiac enzymes but non-obstructive coronary arteries or MINOCA, the next steps in management were unclear. Implementing CMR in this diagnosis has helped to elucidate an underlying etiology clarifying if a patient has myocarditis, stress cardiomyopathy (Takotsubo), coronary dissection, or coronary spasm (22, 91). CMR’s strengths in comprehensively evaluating the myocardium and its pathophysiologic health (e.g., looking for edema, inflammation, and fibrosis), as well as other cardiac structures like the coronary arteries, elevate its importance in being used early in the patient presenting with chest pain.

In the non-acute chest pain setting, CMR is exceptional in identifying small vessel ischemia using vasodilator perfusion. In newer methods, myocardial perfusion may be quantified, and endocardial-to-epicardial myocardial blood flow ratios assessed. Dr. Noel Bairey Merz, a Professor of Medicine at the Cedars Sinai Heart Institute, and others demonstrated the utility of CMR in evaluating women with chronic chest pain who have non-obstructive coronary arteries (22). With a diagnosis of small vessel disease, clinicians then have a therapeutic target; whereas, in the past, without a definitive diagnosis, treatment was directed as a “best guess,” or worse yet, women were told that their chest pain was non-cardiac.

Diagnosing epicardial coronary artery disease in women has also not been straightforward. Exercise treadmill has a notoriously high false-negative rate in women; nuclear stress testing may yield false positive or equivocal results with breast attenuation. However, CMR stress testing provides a comprehensive assessment of myocardial ischemia, independent of a woman’s body mass index or body habitus (92).

Another field that CMR has changed is that of cardio-rheumatology. Rheumatological disorders such as systemic lupus erythematosus, dermatomyositis, and polymyositis affect women greater than men (e.g., 90% of patients with lupus are women between the ages of 15 and 45 years of age). Again, the ability to discern when the heart is actively affected helps guide the management of the rheumatological patient. Newer parametric mapping methods now offer the ability to serially follow patients, even without contrast, to monitor patients’ response to therapy (93, 94).

Cardio-oncology and a host of therapeutic-related adverse events affect both men and women. However, breast cancer cardio-toxicities have mainly utilized CMR effectively to monitor anthracycline toxicities. Active research is ongoing, using CMR to monitor chemo- and immunotherapeutic toxicities, parametric mapping, and myocardial strain. Beyond identification of cardiomyopathy, CMR may also detect therapy-related acute myocarditis. In women who have undergone chest irradiation and are at risk for premature atherosclerosis, CMR provides a comprehensive assessment of ischemic heart disease using pharmacologic or exercise stress testing (95, 96).

The aforementioned uses of CMR are not meant to comprise an exhaustive list but rather demonstrate a few common examples in which CMR has impacted women’s cardiovascular health. Research and clinical translation are ongoing in a multitude of disease processes. MRI pulse sequences and technology are continuously developing, and new CMR applications are constantly evolving.

It is of particular relevance to highlight the role of CMR in improving cardiovascular care since women are under-represented in clinical trials (97–99), limiting biological understanding and contributing to health inequities, social injustice (99), and impacting their health directly as the state-of-the-art treatments and recommendations have been historically mainly male-oriented (100). Within CMR clinical studies and trials, dedicated emphasis in understanding sex as a biologic variable also needs additional attention and should be a goal for current and future studies.

## Conclusion

While there have been challenges to entering and remaining in the field, recent data show that women have become an integral part of the cardiovascular imaging workforce. However, additional work remains to support and increase women’s representation in cardiovascular imaging, academia, and multi-disciplinary societies. Recognition of the need for diversity is more widespread, as well as recognition of the need to better support women in all career stages. For example, women’s representation on moderator and speaking panels requires additional mindfulness and work on the part of the organizers, not simply to fulfill a quota but to add to the depth, breadth, and richness of the meetings as there are qualified women who have valuable and unique expertise to share in the scientific sessions. Similarly, active women’s participation in leadership roles within professional societies adds valuable insights and diversity to the growing field. It should be restated clearly that the entire effort to help engage women in cardiology and radiology is not only fair and proper but also enhances the field—increasing the resilience and level of care.

There has been significant progress in advancing cardiovascular magnetic resonance and cardiovascular imaging. Translational applications of cardiovascular imaging make a difference in cardiovascular diagnosis, management, and prognosis. While the initial involvement of women professionals was low, the engagement of creative, thoughtful physicists, engineers, cardiologists, and radiologists within the field has grown and continues to grow. At this moment, we are just beginning to bend the curve. However, sustained vigilance and creative effort will ensure that the future of women in cardiovascular imaging is hopeful and bright.

## Author contributions

LS-G designed the project. LS-G, NA, JS, SR, YH, VF, KT, NS, PP, CB-D, LB, SM, KO, JS-M, and WB discussed the content, wrote the manuscript, reviewed, and approved the final version.

## Footnotes

1 Sierra-Galan LM, Estrada-Lopez E, Ferrari VA, et al. *Worldwide Variation in Cardiovascular Magnetic Resonance Practice Models.* (In Review).
