# Supplementary material for: Women physicians in cardiovascular magnetic resonance: Past, present, and future
Source: Front Cardiovasc Med. 2023 Jan 4;9:984326. doi: 10.3389/fcvm.2022.984326 (PMC9848434; doi:10.3389/fcvm.2022.984326)
Supplement: Supplementary file 1 [file Data_Sheet_1.docx]

**Supplementary Material**

**Figure 2. Top 20 countries of origin of surveyed respondents (n=1071)**

The top 20 countries with the most respondents are shown. The data from all other respondent countries are not displayed in the graph. The detailed description of the responders and their gender (within parentheses) were as follows, for USA 309 (F:73/M:236), UK 112 (F:46/M:66), Brazil 85 (F:21/M:64), Germany 62 (F:15/M:47), Japan 50 (F:7/M:43), Canada 42 (F:18/M:24), India 31 (F:14/M:17), the Netherlands 30 (F:1/M:29), Switzerland 22 (F:7/M:15), Italy 22 (F:9/M:13), China 18 (F:7/M:11), Australia 18 (F:6/M:12), Spain 18 (F:7/M:11), South Africa 17 (F:6/M:11), Malaysia 15 (F:8/M:7), Mexico 15 (F:7/M:8), Sweden 12 (F:6/M:6), Egypt 10 (F:7/M:3), Greece 10 (F:2/M:8), Singapore 10 (F:4/M:6), Argentina 9 (F:3/M:6), Thailand 8 (F:6/M:2), Hong Kong 8 (F:4/M:4), Saudi Arabia 8 (F:2/M:6), France 8 (F:0/M:8), Turkey 8 (F:0/M:8), Norway 7 (F:4/M:3), Ireland 7 (F:1/M:6), Portugal 6 (F:1/M:5), Colombia 6 (F:2/M:4), Austria 6 (F:2/M:4), Denmark 5 (F:2/M:3), Hungary 5 (F:1/M:4), New Zealand 5 (F:4/M:1), Czech Republic 4 (F:1/M:3), Indonesia 4 (F:3/M:1), Lithuania 4 (F:2/M:2), Romania 4 (F:3/M:1), Philippines 3 (F:3/M:0), Chile 3 (F:1/M:2), Belgium 3 (F:0/M:3), Uruguay 3 (F:2/M:1), Kuwait 3 (F:2/M:1), Korea, South 3 (F:1/M:2), Finland 2 (F:1/M:1), El Salvador 2 (F:2/M:0), Republic of Korea 2 (F:1/M:1), United Arab Emirates 2 (F:0/M:2), Qatar 2 (F:0/M:2), Bangladesh 2 (F:0/M:2), Algeria 2 (F:1/M:1), Slovakia 1 (F:0/M:1), Poland 1 (F:0/M:1), Panama 1 (F:1/M:0), Pakistan 1 (F:0/M:1), Myanmar 1 (F:1/M:0), Morocco 1 (F:1/M:0), Ecuador 1 (F:0/M:1), Andorra 1 (F:0/M:1), Venezuela 1 (F:0/M:1), Russia 1 (F:1/M:0), Oman 1 (F:0/M:1), Mongolia 1 (F:0/M:1), Lebanon 1 (F:0/M:1), Vietnam 1 (F:0/M:1), Nicaragua 1 (F:1/M:0), Iran (Islamic Republic of) 1 (F:1/M:0), Georgia 1 (F:1/M:0), Kazakhstan 1 (F:0/M:1), Monaco 1 (F:1/M:0).
